# A Comparison Between Computer-Assisted Self-Triage by Patients and Triage Performed by Nurses in the Emergency Department

**DOI:** 10.7759/cureus.14002

**Published:** 2021-03-19

**Authors:** Sachin Trivedi, Jessica Littmann, James Stempien, Puneet Kapur, Rhonda Bryce, Martin Betz

**Affiliations:** 1 Emergency Medicine, Royal University Hospital, University of Saskatchewan, Saskatoon, CAN; 2 Orthopedics, University of Manitoba, Winnipeg, CAN; 3 Emergency Medicine, University of Saskatchewan, Saskatoon, CAN; 4 Emergency Medicine, Jim Pattison Children's Hospital, University of Saskatchewan, Saskatoon, CAN; 5 Clinical Research Support Unit, University of Saskatchewan, Saskatoon, CAN; 6 Emergency Medicine, Sunnybrook Health Sciences Centre, Toronto, CAN

**Keywords:** emergency medicine, triage, over-crowding, hospital administration

## Abstract

Background and objective

Emergency departments (EDs) often find the number of arriving patients exceeding their capacity and find it difficult to triage them in a timely manner. The potential risk to the safety of patients awaiting assessment by a triage professional has led some hospitals to consider implementing patient self-triage, such as using kiosks. Published studies about patient self-triage are scarce and information about patients’ ability to accurately assess the acuity of their condition or predict their need to be hospitalized is limited. In this study, we aimed to compare computer-assisted patient self-triage scores versus the scores assigned by the dedicated ED triage nurse (TN).

Methods

This pilot study enrolled patients presenting to a tertiary care hospital ED without ambulance transport. They were asked a short series of simple questions based on an algorithm, which then generated a triage score. Patients were asked whether they were likely to be admitted to the hospital. Patients then entered the usual ED system of triage. The algorithm-generated triage score was then compared with the Canadian Triage and Acuity Scale (CTAS) score assigned by the TN. Whether the patients actually required hospital admission was determined by checking their medical records.

Results

Among the 492 patients enrolled, agreement of triage scores was observed in 27%. Acuity was overestimated by 65% of patients. Underestimation of acuity occurred in 8%. Among patients predicting hospitalization, 17% were admitted, but the odds ratio (OR) for admission was 3.4. Half of the patients with cardiorespiratory complaints were correct in predicting the need for hospitalization.

Conclusion

The use of a short questionnaire by patients to self-triage showed limited accuracy, but sensitivity was high for some serious medical conditions. The prediction of hospitalization was more accurate with regard to cardiorespiratory complaints.

## Introduction

Emergency department (ED) overcrowding is a problem experienced in hospitals worldwide, including North America [1,2]. It results in prolonged waiting times for patients arriving in EDs, increasing the risk of poor clinical outcomes for the sickest patients [3]. Triage has long been used in situations where emergent demand for care overwhelms the availability of appropriately trained healthcare personnel or equipment. All busy EDs now utilize triage scoring systems to help decide which patients are at higher risk of poor outcomes among those who are waiting to be assessed and treated. Triage categorization of arriving patients is usually performed by specially trained registered nurses, though other models of triage also exist, such as those incorporating physicians [4]. Generally, scoring systems are supported by clinical studies that show them to be moderately valid and reliable in predicting illness severity or the need for admission to hospital [5].

Despite the addition of computerization to the process, overcrowding has led to patients in many departments having to wait to be triaged [6]. Risks to patient safety have led some EDs, including the study ED, to put in place processes whereby arriving patients are quickly screened for serious conditions while awaiting formal triage [7].

The idea of enabling caregivers or patients to triage themselves has been proposed to improve EDs' throughput and potentially improve triage performance [8,9]. In particular, the most important reason to implement self-triage is to enhance the delivery of patient-centered care while possibly improving patient safety. Despite the appeal of the idea of patient self-triage, very few studies exist in the medical literature about its feasibility and clinical outcomes.

We hypothesized that patients, or their caregivers, would be able to assign an appropriate triage category by utilizing a computer-based algorithm. The accuracy of patient self-triage has not been previously measured, but it has been reported that ED patients can predict the need for hospitalization relatively well [10]. This pilot study was designed to measure the ability of patients to predict the need for hospitalization and to compare computer-assisted self-triage scores with the scores assigned by the dedicated ED triage nurse (TN).

## Materials and methods

This was a prospective, observational pilot study conducted in the ED of the Royal University Hospital, a tertiary care center in Saskatoon, Canada, serving a population of about 400,000 people. Its annual census is approximately 50,000. Operational approval for this study was obtained from the Saskatoon Health Region. Due to the quality-assurance nature of the study, the University of Saskatchewan’s Biomedical Research Ethics Board granted a letter of exemption from ethics review.

The study population was a convenience sample of patients arriving at the ED on their own, with family or caregivers. Patients brought by ambulance, those not speaking or reading English, and those less than 16 years of age were excluded. Subjects were enrolled only when a specified researcher (JL) was present in the ED, which included weekdays and weekends, extending into evenings on occasion, but not after midnight.

The primary outcome measure was hospitalization, which was compared to patients’ predictions for their need to be admitted to the hospital. The secondary outcome was an algorithm-generated self-triage score (AGST), arrived at by a computer algorithm utilizing patients’ answers to a simple questionnaire alone, which was compared to the Canadian Triage and Acuity Scale (CTAS) score assigned by a TN.

Prospective subjects were identified upon their arrival at the ED by the researcher and given a brief verbal outline of the study. A more detailed description, including an outline of the basic workings of the triage system, was provided in the form of text on an electronic tablet (iPad, Apple Corp, Cupertino, CA). The researcher remained present to answer questions and assist with tablet use. Informed of the study’s intent and process, patients or their caregivers gave consent by clicking a button on the tablet’s screen, leading into the questionnaire. The algorithmic questionnaire was completed by the patient or caregiver on the tablet by touching the screen to choose the answer most appropriate to their situation. Subjects were asked to predict whether they would require admission to the hospital. At the end of the questionnaire, the AGST score was assigned, based on the answers provided. Subjects were blind to the AGST.

Subsequently, the patient underwent the standard triage process, being assessed by a TN, who was also blinded to the AGST score. A CTAS score was then assigned in the usual way.

**Figure 1:**
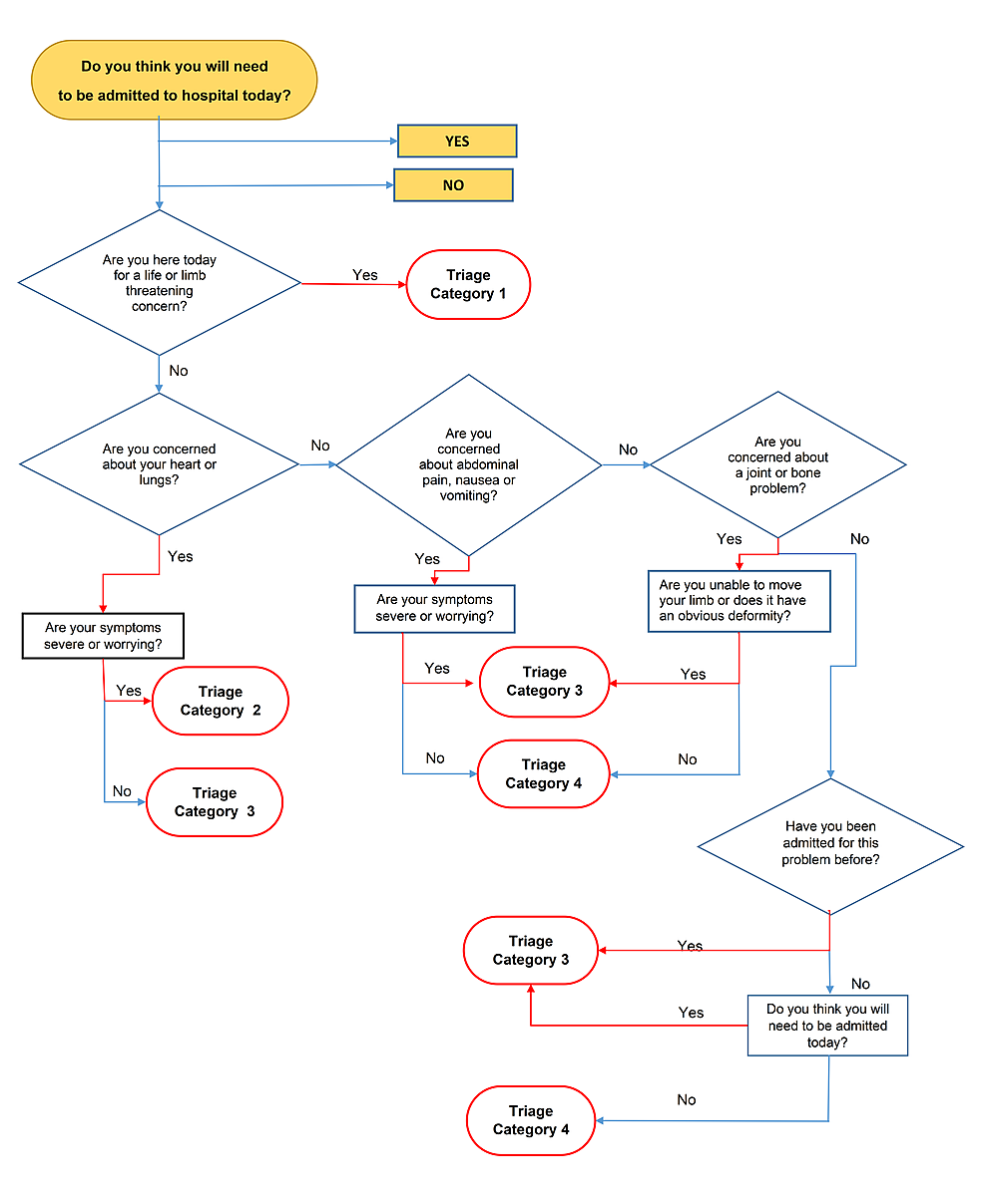
Algorithmic representation of prediction for admission and self-triage questionnaire

The questionnaire consisted of a series of “Yes/No” answers displayed electronically in algorithmic sequence, depending on the answers provided (Figure 1). This algorithm was transcribed into an electronic form by utilizing a software platform (FluidSurvey, SurveyMonkey Canada Inc., Ottawa, Canada). The answers were transmitted from the tablet wirelessly for storage on an electronic database together with patient identifiers. The questionnaire was developed *a priori* by the authors without referral to previously published algorithms. A fundamental premise in its development was that the questionnaire should be short, which could be easily answered by patients with limited health literacy without any outside assistance, and directed toward the most common presenting complaints. The aim was to start with a short, simple questionnaire, rather than a comprehensive health survey that would present completion difficulties for a large number of arriving patients.

It should be noted that the AGST algorithm did not allow for the prediction of category 5 scores separate from category 4 scores. The official triage score was obtained from the patient’s chart and entered into the database. Whether the patient was ultimately discharged from the ED or admitted to the hospital was gleaned from the patient’s electronic medical record. The data was entered into a Microsoft Office Excel electronic worksheet (Microsoft Corporation, Redmond, WA) for analysis.

Basic descriptive statistics were used to summarize age, sex, and presenting complaints of participants. The proportion of patients at each level of acuity (1, 2, 3, 4/5) was calculated for both the AGST and TN CTAS approaches. The proportion of patients with the agreement between their AGST scores and TN CTAS scores, as well as the proportions of AGST scores respectively underestimating and overestimating TN CTAS, were calculated. Similar assessments were made for predetermined subgroups based on age and sex, and comparisons were made across categories using Chi-square testing. For analysis, TN CTAS scores of 4 and 5 were combined into a single triage level to allow for comparison with the AGST’s lowest category. 

Prediction of hospital admission was compared with the actual admission data utilizing McNemar testing. Odds ratios (OR) with 95% confidence intervals (CI) were calculated for these predictions, both overall and by presenting complaints. Chi-square testing was used to evaluate differences in proportions admitted across scores within the respective triage approaches. Statistical analysis was performed by using the SAS software, v9.4 (SAS Institute, Cary, NC).

## Results

A total of 513 participants were approached for enrolment in the study. After excluding ineligible patients, 492 patients were ultimately enrolled. The mean age of enrolled patients was 47 years with a standard deviation of 22 years. The characteristics of the subjects are summarized in Table 1.

**Table 1:** Subject characteristics GI: gastrointestinal

Characteristics		N (%)
Sex	Male	245 (49.8)
	Female	247 (50.2)
Age, years	<20	56 (11.4)
	20-39	168 (34.1)
	40-59	116 (23.6)
	>60	152 (30.9)
Self-perceived problem	Life-/limb-threatening condition	146 (29.7)
	Heart or lung condition	74 (15.2)
	Abdominal pain, GI upset	89 (18.1)
	Musculoskeletal disorder	73 (14.8)
	Other	110 (22.2)

The most frequent AGST score was category 3 (n=176, 36%) followed by category 1 (n=146, 30%), while the least likely AGST score was category 2 (n=66, 21%) (Table 2). TN CTAS scores are also shown in Table 2; the majority of patients were triaged as CTAS 4/5, while none were triaged as category 1. The proportion of patients whose AGST scores agreed with the TN's scores was 27%. In a majority of participants (65%), the TN CTAS scores were lower than the AGST scores, although the scores were higher in 24% of patients with AGST scores of 4/5 (Table 3).

**Table 2:** Admission to hospital as predicted by subjects and by triage category *Admission data missing for 50 patients (10%) AGST: algorithm-generated self-triage score; CTAS: Canadian Triage and Acuity Scale

Admission as self-predicted and by triage category (n=492)			
		Self-assessed (AGST)	Triage nurse (CTAS)
Triage category		N (%)	N (%)
1		146 (30)	0
2		66 (13)	47 (10)
3		176 (36)	155 (32)
4 or 5		104 (21)	290 (59)
Admission to hospital*		Expected	Actual
		203 (41)	76 (17)

**Table 3:** Agreement between AGST score and TN CTAS score: overall, by age, sex, and triage category *P-values for comparison of agreement categories: age, continuous = 0.43 (ANOVA); age, categorical = 0.53; ^†^p-values for comparison of agreement between sexes = 0.04 AGST: algorithm-generated self-triage score; TN: triage nurse; CTAS: Canadian Triage and Acuity Scale; ANOVA: analysis of variance

Agreement between AGST score and TN CTAS score (n=492)			
	Agree	Overestimate	Underestimate
Overall, n (%)	132 (27)	321 (65)	39 (8)
Age, years (mean)	44.7	47.6	46.9
Age group, n (%)			
<20 years	13 (23)	37 (66)	6 (11)
20-39 years	52 (31)	105 (63)	11 (7)
40-59 years	34 (29)	74 (64)	8 (7)
>60 years	33 (22)	105 (69)	14 (9)
Sex^†^, n (%)			
Male	61 (25)	171 (70)	13 (5)
Female	71 (29)	150 (61)	26 (11)
AGST score, n (%)			
1	0	146 (29.7)	NA
2	9 (13.6)	57 (86.4)	0
3	60 (34.1)	102 (58.0)	14 (8.0)
4 or 5	79 (76)	NA	25 (24.0)
TN CTAS score, n (%)			
1	0	-	-
2	9 (19.1)	21 (44.7)	17 (36.2)
3	60 (38.7)	73 (47.1)	22 (14.2)
4 or 5	79 (27.2)	211 (72.8)	NA

**Figure 2:**
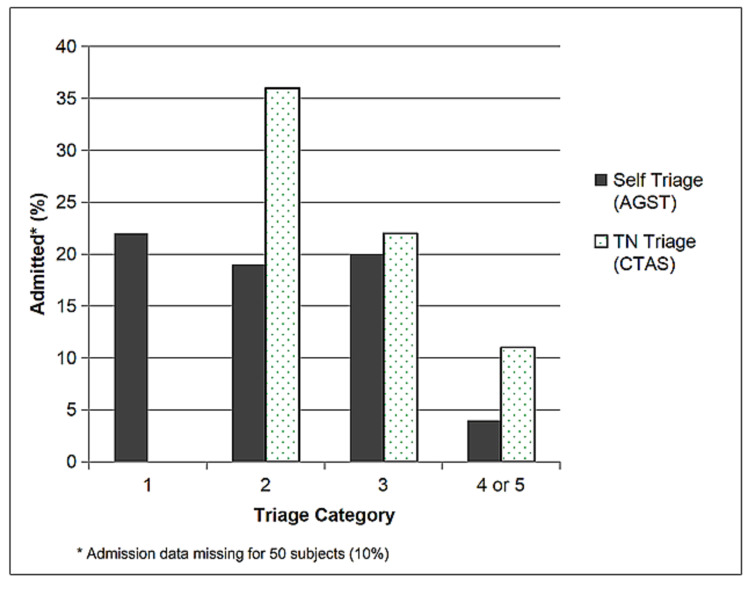
Admission rate by self-triage and triage nurse category AGST: algorithm-generated self-triage score; CTAS: Canadian Triage and Acuity Scale

Figure 2 presents a comparison between the admission rate by self-triage and TN category.

## Discussion

The impetus to look at patient self-assessment arises from the broadly experienced problem of prolonged wait times for patients attending EDs [1,11]. The gap between demand and existing resources in emergency medical care has been increasing for over at least two decades in North America [2]. With a view to improving the safety of patients waiting for definitive care, evermore sophisticated triage systems have been developed to prevent adverse health outcomes in patients.

Very few studies about patient self-triage have appeared in the medical literature, despite it seeming a logical extension of patient-centered healthcare. It is patients, and their families or caregivers, who decide to come to an ED in the first place, rather than waiting for an appointment with a physician who knows them. This already is a form of self-triage, especially since ED overcrowding and excessive wait times are well known to patients. Primary care physicians have identified potentially ill patients who express reluctance to go to an ED due to expected prolonged waiting times (personal communication, Ver Beek 2018). 

Comparing patients’ predictions of the need for hospitalization to actual admissions is a measure of the validity of the self-triage process. A majority of patients overestimate the seriousness of their conditions; among participants who expected admissions, only 27% were actually admitted. However, only 11% of participants not expecting admission were hospitalized. The OR for a patient predicting admission actually being admitted was 3.4. This is less than the OR of 10.68 found by Miyamichi in a Japanese patient cohort [10]. This may reflect a difference in patient populations or in questionnaire design. In our cohort, among patients with heart- or lung-related complaints, 47% of those expecting admission were subsequently admitted, suggesting that clinically important information can be obtained quickly through self-triage, potentially reducing the risk for adverse outcomes in some patients awaiting care.

Our results show a relatively poor agreement between AGST scores by patients and CTAS scores assigned by a TN. The large discrepancies mainly reflect patients’ overestimation of the severity of their illnesses or injuries. The chances of undertriage were low at 8% overall. If self-triage is viewed as a type of screening tool, high sensitivity at the expense of low specificity is typical, and necessarily preferable to the reverse. The rate of undertriage was not zero, but very low: three patients with AGST scores of 4 were given CTAS scores of 2, as were eight patients with AGST scores of 3.

Health self-assessment has been studied in various non-acute, outpatient care settings. For example, a correlation was found in an elderly population between self-assessed health and medium- to longer-term health outcomes such as mortality and hospitalization [12]. In the acute care field, very few studies have been published, and they have mainly focused on specific specialties. Sensitivities for serious conditions are highly varied. A study of patients attending a specialty eye hospital ED, which utilized a complex questionnaire, showed good sensitivity (0.89) for having a condition of high urgency, which was comparable to an assessment by a trained “triage assistant” [13]. In another European study, patients attending a gynecologic hospital with pelvic pain demonstrated a low sensitivity (44%) for predicting a life-threatening condition when completing a questionnaire [14]. In a pediatric hospital, parents’ assessment of their young children being seriously ill was as sensitive as a physician’s gut feeling about a serious illness being present (>90%) [15]. A web-based, algorithmic self-assessment tool used by 294 parents of ill children identified 87.4% of children to be at high risk for more serious illness, missing one of the 15 children who were seriously ill (sensitivity: 93%, specificity: 12.9%).

In a Japanese study of self-assessment by patients visiting a general hospital ED [10], patients, or their caregivers, were asked on arrival (1) to rate the acuity of their disorder, (2) the urgency for treatment, and (3) to predict the need for the hospital admission. The rate of admission increased with an increasing acuity score (1-10): 50% were admitted with a score of 9. Patients who felt they required only a prescription, without testing, had a very low hospitalization rate (0.25%). The authors concluded that patient self-assessment was a valuable adjunct to the triage process, despite noting that patients’ self-assessed need to be urgently treated may have been inflated by the belief that an expressed higher urgency might result in a shorter wait time. The generalizability of this self-triage study from Japan is questionable, although self-interested manipulation is likely to occur with a self-assessment scoring system anywhere.

Although our hypothesis that patients can accurately self-triage was not fully supported by the study outcome, the likelihood of being assigned a more acute CTAS score was high in patients with more acute self-assigned scores. Patients who self-identified with a very simple algorithm as non-urgent were unlikely to require admission or immediate intervention, whereas patients with self-identified, more serious cardiorespiratory complaints were more likely to require admission than the average patient.

Triage is inherently imprecise. Accuracy and validity improve when time is taken to obtain more information about the patient: especially more history, but also assessing physical findings such as vital signs, performing an ECG, or measuring capillary blood glucose. Overtriage remains necessary to maintain adequate sensitivity. That the differential diagnosis of any symptom includes a life- or limb-threatening condition is understood by patients. They just may not be as capable as healthcare providers to determine their risk of having such a condition, hence the large proportion of subjects who self-triaged as category 1 in this study.

Future research analyzing how ED patients estimate their risk for a serious condition may be beneficial in improving the triage process to enhance precision while minimizing the expenditure of time and resources. The increasing use of machine learning to analyze large databases for empiric correlation between patient presentations and outcomes may lead to more insights into this key aspect of self-triage.

There has been some debate about the extent of the value of triage systems [16,17]. The search for improved methods of safely dealing with the crush of patients continues. It may include self-triage together with “quick-look” triage by a TN.

Limitations

The study population was a convenience sample of patients visiting the ED. No patients visiting after midnight were included, and this may have possibly introduced bias into the process. The questionnaire, although designed to be self-administered using a computer touch screen, was found difficult to use by some patients and required assistance by a medically knowledgeable person, which may have influenced answers.

Our findings of the overestimation of CTAS acuity could be partly due to the questionnaire design. The algorithm was intentionally kept short and simple. The number of chief complaints was limited to those that were life-/limb-threatening, and those related to heart and lungs, and abdomen and bones/joints. A number of presenting problems, especially neurological and mental health complaints, were not included in the questionnaire, except under the final, non-specific, catch-all query.

The absence of data on patient disposition in 52 (11%) patients appears to have affected the admission data. The remote possibility that those patients were all admitted would have resulted in an admission rate of 26% rather than the current 17%. However, it is likely that this data was missing due to those patients leaving the ED before being fully medically assessed.

Although the primary outcome was a comparison against a gold standard that could be described as a patient outcome, the gold standard for the secondary outcome of AGST triage score comparison was the CTAS score by the TN. Triage systems are imperfect, with published validity measures being rather variable, especially when assessed from outside the system [5,18]. Actual patient illness acuity, as determined by clinical outcome, or as judged by the emergency physician, was not utilized. Comparing patient self-triage to TN-assigned CTAS scores, rather than actual patient outcomes, is more a measure of the reliability than the validity of the self-triage process.

## Conclusions

EDs constantly seek to decrease wait times and protect patients against adverse outcomes while waiting. Asking patients to triage themselves has been proposed as a method to address safety concerns for patients waiting to see a TN and to improve the throughput of patients. Very few studies have looked at the accuracy of patient self-assessments compared to those performed by healthcare workers, especially in the acute care setting. This study revealed a relatively poor agreement (about 20%) between patient-assigned and TN-assigned triage categories. However, only a small minority of patients underestimated the acuity of their condition, when compared to a standard triage system. Although the prediction of the need for admission to the hospital was poor (17%), patients who believed they required admission were 3.4 times more likely to be admitted than those who did not. Patients with the lowest acuity self-triage score had a rate of admission less than 5% compared to more than 20% for those with higher scores. A subgroup of patients with cardiorespiratory problems was more accurate in predicting the need for admission (47%).
